# O_2_-Filled Swimbladder Employs Monocarboxylate Transporters for the Generation of O_2_ by Lactate-Induced Root Effect Hemoglobin

**DOI:** 10.1371/journal.pone.0034579

**Published:** 2012-04-04

**Authors:** Takahiro Umezawa, Akira Kato, Maho Ogoshi, Kayoko Ookata, Keijiro Munakata, Yoko Yamamoto, Zinia Islam, Hiroyuki Doi, Michael F. Romero, Shigehisa Hirose

**Affiliations:** 1 Department of Biological Sciences, Tokyo Institute of Technology, Yokohama, Japan; 2 Department of Physiology and Biomedical Engineering, Mayo Clinic College of Medicine, Rochester, Minnesota, United States of America; 3 Ushimado Marine Laboratory, Okayama University, Shimonoseki Marine Science Museum, Setouchi, Japan; 4 Shimonoseki Academy of Marine Science, Shimonoseki, Japan; Roehampton University, United Kingdom

## Abstract

The swimbladder volume is regulated by O_2_ transfer between the luminal space and the blood In the swimbladder, lactic acid generation by anaerobic glycolysis in the gas gland epithelial cells and its recycling through the rete mirabile bundles of countercurrent capillaries are essential for local blood acidification and oxygen liberation from hemoglobin by the “Root effect.” While O_2_ generation is critical for fish flotation, the molecular mechanism of the secretion and recycling of lactic acid in this critical process is not clear. To clarify molecules that are involved in the blood acidification and visualize the route of lactic acid movement, we analyzed the expression of 17 members of the H**^+^**/monocarboxylate transporter (MCT) family in the fugu genome and found that only MCT1b and MCT4b are highly expressed in the fugu swimbladder. Electrophysiological analyses demonstrated that MCT1b is a high-affinity lactate transporter whereas MCT4b is a low-affinity/high-conductance lactate transporter. Immunohistochemistry demonstrated that (i) MCT4b expresses in gas gland cells together with the glycolytic enzyme GAPDH at high level and mediate lactic acid secretion by gas gland cells, and (ii) MCT1b expresses in arterial, but not venous, capillary endothelial cells in rete mirabile and mediates recycling of lactic acid in the rete mirabile by solute-specific transcellular transport. These results clarified the mechanism of the blood acidification in the swimbladder by spatially organized two lactic acid transporters MCT4b and MCT1b.

## Introduction

The swimbladder is a gas-filled internal organ that controls the body buoyancy of teleost fish, help them to stay at a chosen water depth without wasting energy. The gas in swimbladders is composed primarily of O_2_ [1], [2], and the swimbladder volume is regulated by O_2_ transfer between the luminal space of the swimbladder and the blood [3]. Although the O_2_ partial pressures in the swimbladders of living fish are much higher than those in the circulating blood and the surrounding water [2], O_2_ can be transported against the gradient as a result of the Root effect [4]–[6] (Fig. S1A). This effect involves a markedly reduced capacity of fish hemoglobin to bind O_2_ at low pH [7], [8]. Hemoglobin molecules can thus act as acid-controlled molecular oxygen pumps that deliver O_2_ against a high oxygen concentration gradient to the swimbladder. Therefore, local blood acidification in the swimbladder is essential for luminal O_2_ secretion.

The swimbladder is composed of 3 functional components: the oval gland, the gas gland, and the rete mirabile (Fig. S1B). The oval gland, situated on the dorsal side of the swimbladder wall, facilitates O_2_ movement from the lumen of the swimbladder into the blood and reduces the swimbladder volume [9], [10]. The gas gland is located on the ventral side of the swimbladder wall and consists of a thick epithelial layer of gas gland cells and capillaries. Gas gland cells acidify the blood by secreting lactic acid, thus stimulating O_2_ release into the lumen, and thereby increasing the swimbladder volume [11]. Local blood acidification in the gas gland is maintained by the rete mirabile, which consists of a number of arterial and venous capillaries that enable countercurrent blood flow [12], [13] (Fig. S1C, D). Studies by others using cannulated swimbladders [14] and isolated gas gland cells [15] have established that (i) acid secretion largely depends on glucose levels in the blood or media [14], [15]; (ii) although gas gland cells exist under hyperoxic conditions, the gas gland largely secretes lactic acid; and (iii) this acid secretion is not inhibited by cyanide [15]. These facts indicate that anaerobic glucose metabolism is predominant in gas gland cells and that lactic acid secretion is important for blood acidification [11], [14], [15].

In mammals, a family of H^+^/monocarboxylate transporters (MCTs; Slc16) has been shown to play a significant role in lactate metabolism [16], [17]. The MCT family consists of 14 members with 12 transmembrane spans, which mediate H^+^-coupled cotransport of monocarboxylates (e.g., lactate, pyruvate, and ketone bodies) [17]. In this study, therefore, we sought to ascertain whether MCT family members in the swimbladder are involved in the secretion and recycling of lactate that acidify the blood, which in turn promotes unloading of O_2_ from hemoglobin. Taking advantage of the genome availability of *Takifugu rubripes* [18], we developed a model of lactate transfer by 2 MCTs in the gas gland and rete mirabile. An unexpected finding was that MCT1b was expressed in the arterial capillaries but not the venous capillaries of the rete mirabile. This fact is incorporated into our model and helps to better explain the “Root effect.”

## Results

### Histological analyses of the fugu swimbladder

**Figure 1:**
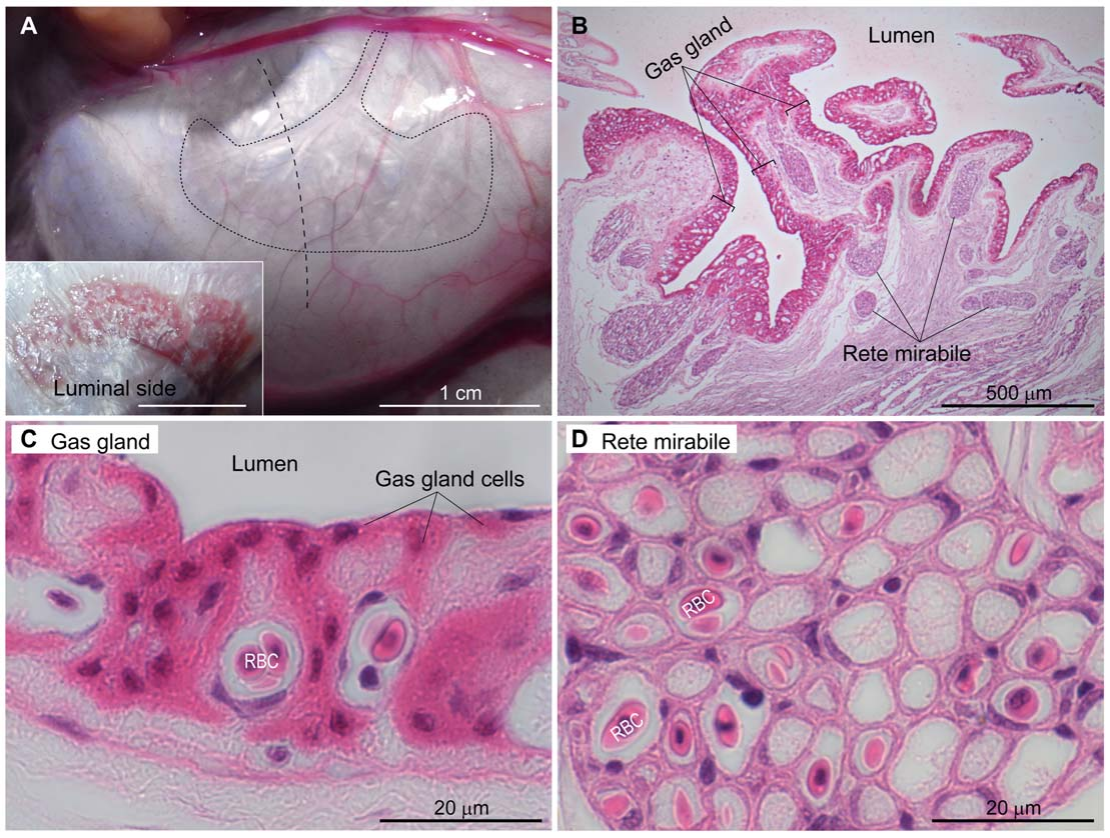
Histology of the fugu swimbladder. A, A picture of the ventral wall of the swimbladder of fugu (*Takifugu rubripes*). The gas gland and rete mirabile exist in the area surrounded by the dotted line. Hematoxylin/eosin-stained images (B–D) are cross-section views cut along a vertical dotted line. B, Paraffin-embedded sections of the ventral wall of the fugu swimbladder were stained with hematoxylin and eosin. C, D, Higher magnification images of the gas gland (C) and rete mirabile (D). RBC, red blood cell.

**Figure 2:**
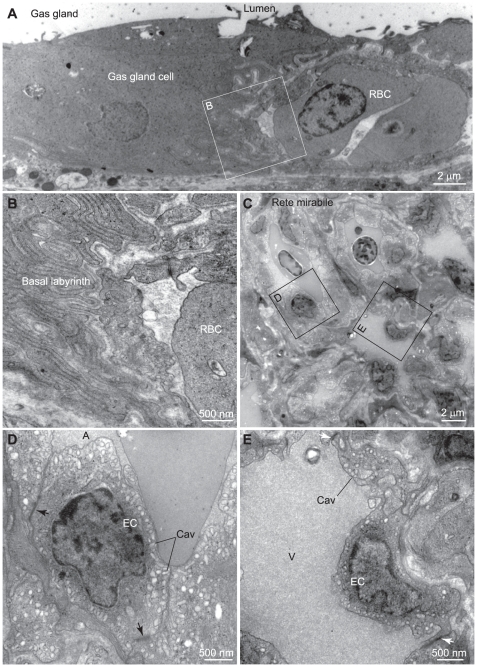
Transmission electron microscopy of fugu gas gland and rete mirabile cells. A, Transmission electron micrograph of a gas gland cell. B, Higher magnification image of the basolateral side of the gas gland cell. C, Transmission electron microscopy of the rete mirabile. D, E, Higher magnification images of the endothelial cells of arterial (D) and venous (E) capillaries in the rete mirabile. RBC, red blood cell; A, arterial capillary; V, venous capillary; EC, endothelial cell; Cav, caveolae-like structure.

The ventral wall of the fugu swimbladder was fixed (Fig. 1A), and the sections were analyzed by light and electron microscopy (Figs. 1, 2). The luminal surface of the ventral wall is corrugated and covered with a thick epithelial layer consisting of capillary endothelial cells, blood cells, and gas gland cells that stained strongly with eosin (Fig. 1B, C). Transmission electron microscopy demonstrated that the thick epithelial cells contained few intracellular membrane structures and had tight junctions and a well-developed basal labyrinth (Fig. 2A, B). The gas gland cells were adjacent to capillary endothelial cells (Figs. 1C, 2A) which provided a connection to the rete mirabile bundle of capillaries (Fig. 1B, D). As demonstrated previously by others [19], [20], the rete mirabile consists of both venous and arterial capillaries surrounded by thick and thin endothelium, respectively (Fig. 2C–E), and arranged side by side in countercurrent fashion. The thick and thin endothelial layers have caveolae-like structures in the apical and basolateral sides of the plasma membrane (Fig. 2D, E). These characteristics of gas gland cells and the rete mirabile are similar to those in the European eel *Anguilla anguilla* [19], [20], the toadfish *Opsanus tau* [21], and the smooth toadfish *Tetractenos glaber* [9].

### Identification of fugu MCT genes and their evolutionary history

**Figure 3:**
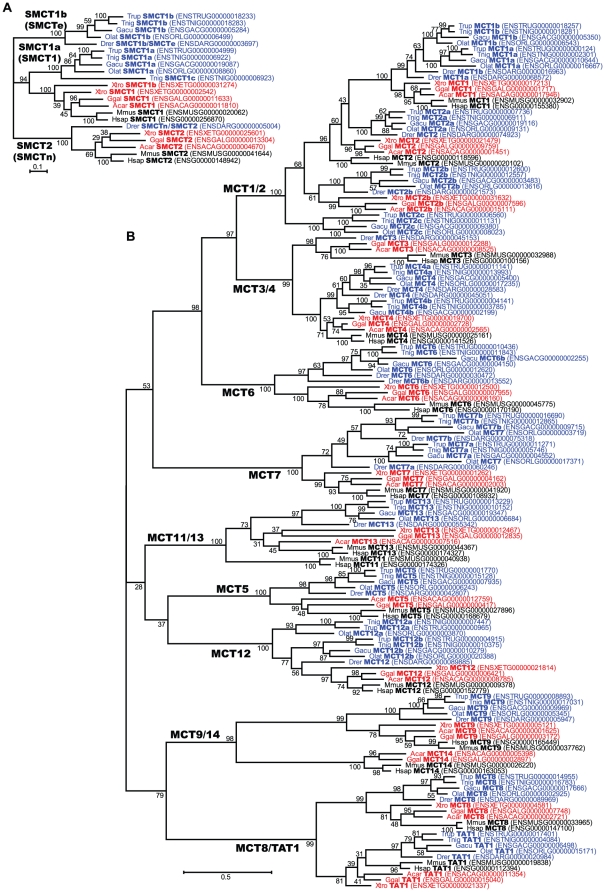
Phylogenetic analyses of fugu MCT and SMCT families. Phylogenetic trees of SMCT (A) and MCT (B) families were constructed using the maximum likelihood method with ClustalW and MEGA4. Numbers indicate bootstrap values and the scale bar represents a genetic distance of amino acid substitutions per site. Trub, fugu; Tnig, *Tetraodon*; Gacu, stickleback; Olat, medaka; Drer, zebrafish; Xtro, *X. tropicalis*; Ggal, chicken; Acar, anole lizard; Mmus, mouse; and Hsap, human.

**Figure 4:**
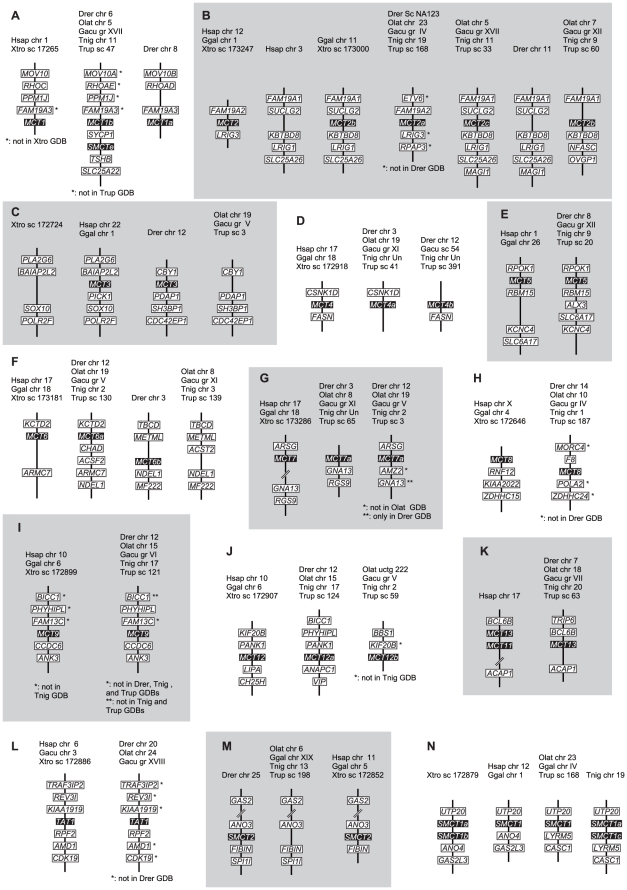
Synteny analysis of *MCT* and *SMCT* gene families. Synteny of neighboring genes of MCTs and SMCTs in the genome databases of human (Hsap), chicken (Ggal), *X. tropicalis* (Xtro), zebrafish (Drer), medaka (Olat), stickleback (Gacu), *Tetraodon* (Tnig), and fugu (Trub) are shown. chr, chromosome; gr, group; sc, scaffold; and uctg, ultracontig.

Database mining identified 16 genes for fugu MCTs (Slc16), one gene for another Slc16 family TAT1 (Slc16a10), an aromatic amino acid transporter, and two genes for Na^+^/monocarboxylate transporters (SMCTs), the other type of lactate transporters belonging to the Slc5 family [22]. We also identified MCT and SMCT gene families from genome databases of several vertebrate species including mammal, bird, reptile, amphibian, and fish. The numbers of the genes and their chromosomal localizations in several species are shown in Tables 1 and 2, respectively. Phylogenetic analyses of predicted amino acid sequences and synteny analysis are shown in Figs 3 and 4, respectively.

**Table 1:** Numbers of MCT and SMCT genes in vertebrate genome databases.

	MCT1	MCT2	MCT3	MCT4	MCT5	MCT6	MCT7	MCT8	MCT9	MCT11	MCT12	MCT13	MCT14	TAT1	SMCT1/e	SMCT2
	SLC16A1	SLC16A7	SLC16A8	SLC16A3	SLC16A4	SLC16A5	SLC16A6	SLC16A2	SLC16A9	SLC16A11	SLC16A12	SLC16A13	SLC16A14	SLC16A10	SLC5A8	SLC5A12
Human	1	1	1	1	1	1	1	1	1	1	1	1	1	1	1	1
Mouse	1	1	1	1	1	1	1	1	1	1	1	1	1	1	1	1
Rat	1	1	1	1	1	1	1	1	1	1	1	1	1	1	1	1
Cow	1	1	1	1	1	1	1	1	1	1	1	1	1	1	1	1
Chicken	1	2	1	1	1	1	1	1	1		1	1	1	1	1	1
Anole Lizard	1	2	1	1	1	1	1	1	1		1	1	1	1	1	1
*X. tropicalis*	1	2		1		1	1	1	1		1	1		1	2	1
Zebrafish	2	2	1	2	1	2	2	1	1		1	1		1	1	1
Medaka	2	3		1	1	1	2	1	1		2	1		1	2	
Stickleback	2	3		2	1	2	2	1	1		1	1		1	2	
*Tetraodon*	2	3		2	1	1	2	1	1		2	1		1	3	
Fugu	2	3		2	1	1	2	1	1		2	1		1	2	

**Table 2:** Chromosomal localization of MCT and SMCT genes in vertebrate genome databases.

Human		Rat		Cow		Chicken		Zebrafish		Medaka		Stickleback	
chr	gene	chr	gene	chr	gene	chr	gene	chr	gene	chr	Gene	chr	Gene
1	MCT1	2	MCT1	3	MCT1	26	MCT1	8	MCT1a	7	MCT1a	12	MCT1a
	MCT5		MCT5		MCT5		MCT5		MCT5		MCT2b		MCT2b
											MCT5		MCT5
						12	MCT2b	23	MCT2b				
								6	MCT1b	5	MCT1b	17	MCT1b
									SMCTe		MCT2c		MCT2c
											SMCTe		SMCTe
12	MCT2	7	MCT2	5	MCT2	1	MCT2a	Un	MCT2a	23	MCT2a	4	MCT2a
	SMCT1		MCT3		MCT3		MCT3				SMCT1		MCT8
			SMCT1		SMCT1		SMCT1						SMCT1
22	MCT3												
X	MCT8	X	MCT8	X	MCT8	4	MCT8	14	MCT8	10	MCT8		
17	MCT4	10	MCT4	19	MCT4	18	MCT4	3	MCT4a	8	MCT4a	11	MCT4a
	MCT6		MCT6		MCT6		MCT6		MCT6b		MCT7a		MCT7a
	MCT7		MCT7		MCT7		MCT7		MCT7a				
	MCT11		MCT11		MCT11								
	MCT13		MCT13		MCT13								
						Un	MCT13	7	MCT13	18	MCT13	7	MCT13
												Un	MCT4b
								12	MCT3	19	MCT6	5	MCT6a
									MCT4b		MCT7b		MCT7b
									MCT6a				MCT12
									MCT7b				
									MCT9				
									MCT12				
10	MCT9	20	MCT9	28	MCT9	6	MCT9			15	MCT9	6	MCT9
	MCT12						MCT12				MCT12a		
		1	MCT12	26	MCT12								
2	MCT14	9	MCT14	2	MCT14	9	MCT14						
6	TAT1	20	TAT1	9	TAT1	3	TAT1	20	TAT1	24	TAT1	18	TAT1
										Un	MCT12b	16	MCT6b
11	SMCT2	3	SMCT2	15	SMCT2	5	SMCT2	25	SMCT2				

chr, chromosome; Un, unidentified.

Fish have two paralogous genes for *MCT1*, *MCT4*, *MCT6*, *MCT7*, and *MCT12*, which may be produced by fish-specific whole-genome duplication. Synteny analysis demonstrated that neighboring genes such as *FAM19A3* (Fig. 4A), *NDEL1* (Fig. 4F), and *GNA13* (Fig. 4G) were also duplicated with *MCT1*, *MCT6*, and *MCT7*, respectively. Fish-specific paralogs such as *MCT4b* of medaka, *MCT6b* of several fishes (Fig. 4F), and *MCT12a* of zebrafish and stickleback may have been deleted in each species. MCT6 may have been duplicated again in stickleback (Fig. 3B). Fishes have three *MCT2* genes, and chicken and *X. tropicalis* have two *MCT2* genes. *MCT2b* of chicken and *X. tropicalis* showed homologous synteny with *MCT2b*/*c* of fishes (Fig. 4B). These results suggest that (i) duplication of *MCT2*(*a*) and *MCT2b* occurred before the separation of fish and tetrapod, (ii) mammals lacked *MCT2b* and retain only one *MCT2*, and (iii) duplication of *MCT2a*/*b* is associated with duplication of neighboring genes such as *FAM19A1*/*2* and *LRIG1*/*3*. Synteny analysis also suggests that *MCT2c* was further created by fish-specific whole-genome duplication (Fig. 4B), and *MCT2c* was deleted again in zebrafish. *MCT3* was deleted in *X. tropicalis* and several fishes (Fig. 4C), but is retained in zebrafish. *MCT11* is similar to *MCT13*, is located next to *MCT13* in mammalian genome (Fig. 4K), and was not found in fish genome databases, suggesting that *MCT11* is a mammalian-specific paralog that was arose by tandem gene duplication. Zebrafish has *SMCTe* and *SMCTn*/*SMCT2*, but the other fishes such as medaka, stickleback, *Tetraodon*, and fugu have *SMCT1* (*SMCT1a*) and *SMCTe* (*SMCT1b*) (Fig. 3A). Tetrapods have *SMCT1* and *SMCT2*, but not *SMCTe*. We could not find good evidence that *SMCTe* (*SMCT1b*) is a fish-specific paralog or tetrapods lost *SMCTe* during evolution. *X. tropicalis* and *Tetraodon* have two tandemly located *SMCT1* genes.

### Expression analyses of the fugu MCT gene family

**Figure 5:**
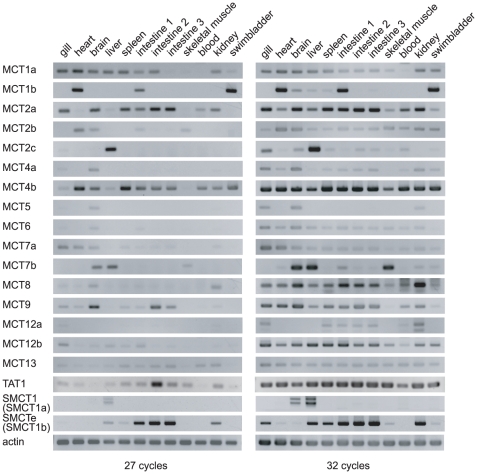
Expression analyses of fugu MCT and SMCT families. Tissue-specific expression analyses of fugu MCT and SMCT families determined by semiquantitative RT-PCR.

To determine the tissue distribution of fugu MCTs and SMCTs, reverse-transcriptase polymerase chain reaction (RT-PCR) analyses in 12 tissues (27 and 32 cycles) were performed (Fig. 5C). MCT1b and MCT4b were highly expressed in the swimbladder (27 cycles). When the cycle number was increased to 32, weak bands were also detected for other MCTs, but the strengths were much weaker than those of MCT1b and MCT4b. No SMCTs were expressed in the swimbladder, although they are expressed in the zebrafish swimbladder [22]. Therefore, we concluded that MCT1b and MCT4b are the dominant lactate transporters in the fugu swimbladder. MCT1b was highly expressed in the heart and the first segment of the intestine as well as the swimbladder. In contrast, MCT4b was broadly expressed in a variety of tissues (e.g., heart, brain, spleen, all segments of the intestine, blood cells, kidney, and swimbladder).

### Cloning of MCT1b and MCT4b

To determine the amino acid sequence of fugu MCT1b and MCT4b, full-length cDNAs were isolated from the swimbladder of *T. rubripes* by RT-PCR. Fugu MCT1b consists of 455 amino acid residues and has 12 predicted transmembrane segments (Fig. S2A). Fugu MCT4b consists of 467 amino acid residues and has 12 predicted transmembrane segments (Fig. S2B). In both cases, the intracellular loop between transmembrane segments 6 and 7 and the C-terminus show low homology between mammalian and fish MCT paralogs, but the N-terminus and most transmembrane segments are well conserved.

### Localization of MCT1b and MCT4b in the swimbladder

**Figure 6:**
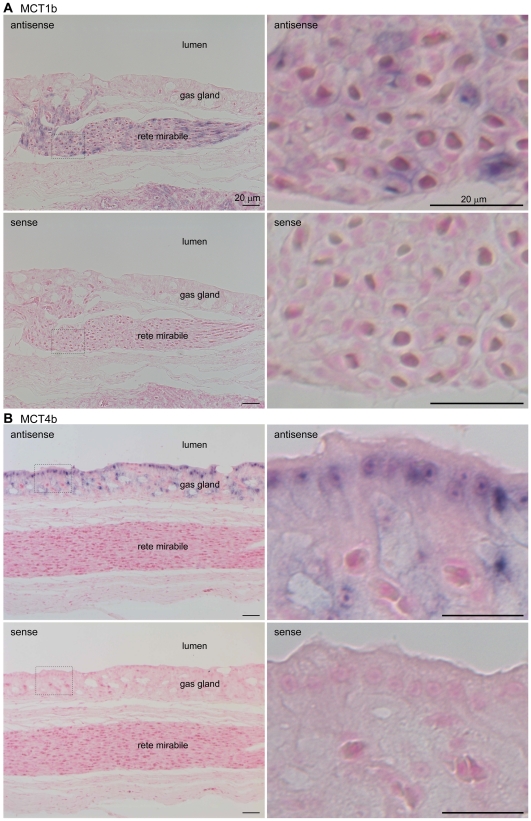
*In situ* hybridization analysis of the distribution of MCT1b and MCT4b mRNAs in the fugu swimbladder. Paraffin-embedded sections from a fugu swimbladder (specifically, the gas gland and rete mirabile) were stained with digoxin-labeled antisense or sense (control) cRNA probes for MCT1b (A) and MCT4b (B). Higher magnification images are shown to the right.

The mRNA distributions of fugu MCT1b and MCT4b in the swimbladder were analyzed by *in situ* hybridization. Digoxigenin (DIG)-labeled antisense cRNA probes for MCT1b and MCT4b produced strong signals in the rete mirabile and gas gland, respectively (Fig. 6), and no significant hybridization was observed with the sense probes (Fig. 6). High-magnification images demonstrated that MCT4b was expressed in gas gland cells.

To identify cells that expressed MCT1b and MCT4b with finer resolution, we developed antisera against fugu MCT1b and MCT4b. The specificity of these antisera was confirmed by immunocytochemical analyses of COS7 cells exogenously expressing MCT1b or MCT4b (Fig. S3).

**Figure 7:**
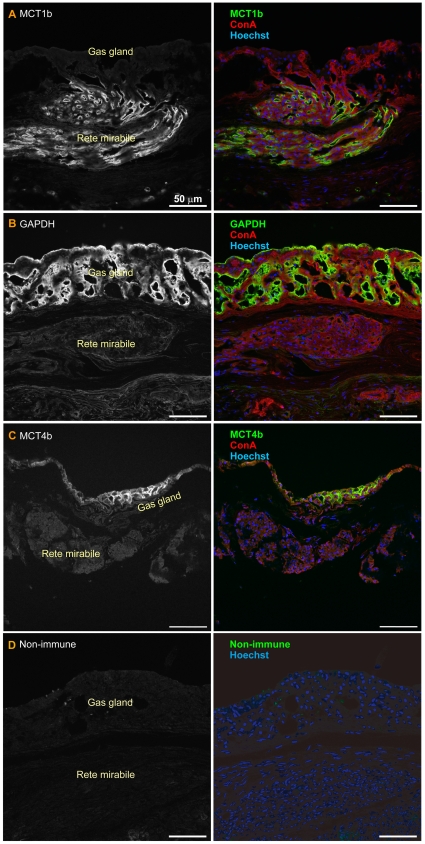
Immunohistochemistry of the fugu swimbladder. Swimbladder sections were stained with anti-MCT1b (A), anti-GAPDH (B), and anti-MCT4b (C) antisera. (D) Control samples stained with nonimmune rabbit serum. The right panels show sections double- or triple-stained with Alexa Fluor 594-labeled concanavalin A (ConA) and Hoechst 33342.

**Figure 8:**
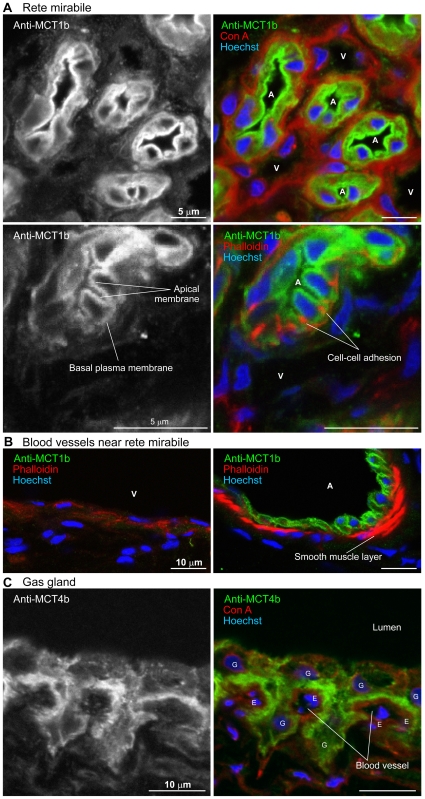
Subcellular localization of MCT1b and MCT4b in the fugu swimbladder. A, High-magnification images of immunohistochemical sections of the rete mirabile stained with anti-MCT1b antiserum. The right panels show sections triple-stained with Hoechst 33342 and/or Alexa Fluor 594-labeled ConA and/or fluorescently labeled phalloidin. A, arterial capillary; V, venous capillary. B, Arterial or venous small blood vessels near the rete mirabile were stained with anti-MCT1b antiserum, Hoechst 33342, and fluorescently labeled phalloidin. A, artery; V, vein. C, High-magnification images of immunohistochemical sections of the gas gland stained with anti-MCT4b antiserum. The right panels show sections triple-stained with Alexa Fluor 594-labeled ConA and Hoechst 33342. E, endothelial cell; G, gas gland cell.

When frozen sections of fugu swimbladder were stained with anti-MCT4b antiserum, strong signals for MCT4b were observed only in gas gland cells (Figs. 7C, 8C). This signal was not observed when the sections were stained with preimmune rabbit serum at the same dilution (Fig. 7D).

When frozen sections of fugu swimbladder were stained with anti-MCT1b antiserum, a strong signal was detected in the rete mirabile (Fig. 7A). This signal was not observed when the sections were stained with preimmune rabbit serum at the same dilution (Fig. 7D). Interestingly, there were no capillaries expressing MCT1b in the gas gland layer (Fig. 7A). The sections were costained with fluorescence-labeled concanavalin A (basal lamina surrounding each capillary), and with fluorescence-labeled phalloidin (actin filaments), which were concentrated around endothelial cell tight junctions and were highly concentrated in the contractile apparatuses of smooth muscle cells. High-magnification images demonstrated that MCT1b was localized in the thick endothelium of arterial capillaries but not in the thin endothelium of venous capillaries (Fig. 8A). Within the endothelial cells of arterial capillaries, both apical and basal membranes were stained with anti-MCT1b antiserum, with the apical membrane showing stronger staining. Actin filaments at cell–cell adhesion sites in arterial capillaries were stained much stronger than those in venous capillaries. Near the rete mirabile, thick endothelium expressing MCT1b were also observed in small arteries surrounded by smooth muscle cells, and thin endothelium lacking MCT1b expression were also observed in small veins lacking a smooth muscle layer (Fig. 8B).

### Immunohistochemical analyses of GAPDH

The finding that MCT1b was highly expressed in the rete mirabile arterial capillaries raised the question of whether MCT1b (a) mediates transendothelial transport of lactic acid or (b) secretes lactic acid generated by the endothelial cells themselves. To answer this question, frozen sections of fugu swimbladder were stained with antibody to the glycolytic enzyme glyceraldehyde 3-phosphate dehydrogenase (GAPDH), whose levels were expected to be highly elevated in cells secreting lactate. Strong signals for GAPDH were observed in gas gland cells only, not in rete mirabile cells (Fig. 7B). This result strongly suggests that (i) the major site of lactic acid production in the swimbladder is gas gland cells, and (ii) MCT1b mediates transendothelial transport of lactic acid in the rete mirabile countercurrent system.

### MCT1b and MCT4b are electroneutral H^+^/lactate cotransporters

**Figure 9:**
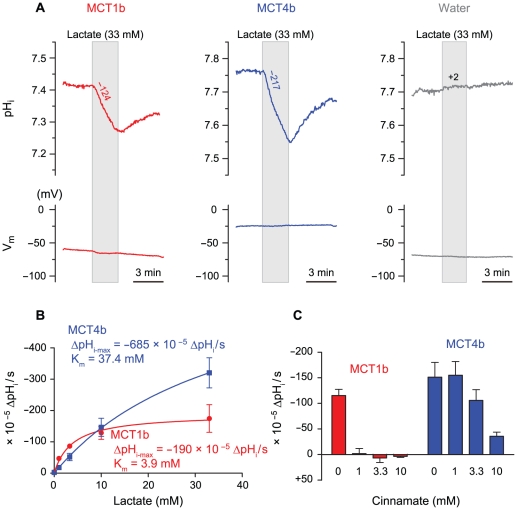
Electroneutral H^+^/lactate cotransport mediated by fugu MCT1b and MCT4b. A, Representative traces of intracellular pH (pH_i_) and membrane potential (V_m_) of oocytes injected with MCT1b (red), MCT4b (blue), or water (gray) are shown. The H^+^/lactate cotransport activities were monitored via pH_i_ changes when 20 mM lactate was added. Gray boxes indicate results during a solution change from 70 mM Cl^−^ ND96 (70-Cl ND96) to 70-Cl ND96 containing 20 mM lactate. Numbers above pH_i_ traces are the initial rates of change in ΔpH_i_/s. B, Michaelis–Menten curves fitted to lactate-elicited ΔpH_i_ in oocytes expressing MCT1b and MCT4b. ΔpH_i_ was measured by addition of 0, 1, 3.3, 10, or 33 mM lactate in the presence of 70 mM Cl^−^. Maximum ΔpH_i_ (ΔpH_i-max_) and the Michaelis–Menten constant (K_m_) are shown. Values are expressed as means ± SE, *n* = 3–12. C, Inhibition of MCT1b and MCT4b by cinnamate (α-cyano-4-hydroxycinnamic acid). In the continuous presence of 0, 1, 3.3, and 10 mM cinnamate, the initial rates of change in ΔpH_i_/s were measured when 10 mM lactate was added. Values are expressed as means ± SE, *n* = 4–12.

The activities of MCT1b and MCT4b were measured using *Xenopus* oocytes injected with fugu MCT1b or MCT4b cRNAs. The intracellular pH was monitored with a pH microelectrode in response to lactate addition (Fig. 9). Water-injected oocytes were used as a negative control. Exposure to 20 mM lactate caused marked reduction of pH_i_ (MCT1b: −153±38×10^−5^ pH units/s, *n* = 7; MCT4b: −314±37×10^−5^ pH units/s, *n* = 6; water: −6±2×10^−5^ pH units/s, *n* = 7) but did not elicit changes in membrane potential (Fig. 9A). Removal of lactate induced recovery of pH_i_. Control oocytes did not show these responses. These results indicate that both MCT1b and MCT4b mediate electroneutral H^+^/lactate cotransport.

### Kinetics of MCT1b and MCT4b

Initial rates of pH_i_ change per second in oocytes expressing fugu MCT1b and MCT4b were measured after addition of 1, 3.3, 10, or 33 mM lactate (Fig. 9B). In media containing 1 or 3.3 mM lactate, dpH_i_/dt values for MCT1b were 1.6–2.6 times larger than those of MCT4b (*P*<0.05, *n* = 3–4). In medium containing 10 mM lactate, ΔpH_i_/s values were similar between MCT1b and MCT4b. In medium containing 33 mM lactate, the dpH_i_/dt values for MCT4b was ∼2 times greater than that of MCT1b (*P*<0.01, *n* = 6–7). Next, lactate steady-state kinetics were calculated based on the Michaelis–Menten equation (Fig. 9B). The ΔpH_i-max_ value of MCT4b for lactate (685±37×10^−5^ ΔpH_i_/s) was 3.6 times higher than that of MCT1b (190±10×10^−5^ ΔpH_i_/s). In contrast, MCT1b showed a ∼10-fold higher affinity for lactate than MCT4b (*K_m_* = 3.9±0.7 mM for MCT1b; and 37.4±2.7 mM for MCT4b) (*P*<0.001, *n* = 5). These results indicate that MCT1b is a high-affinity lactate transporter whereas MCT4b is a low-affinity/high-capacity lactate transporter.

### Differential cinnamate sensitivity of MCT1b and MCT4b

Inhibition of lactate-transport activities by cinnamate was evaluated by measuring ΔpH_i_ in the presence of 10 mM lactate and 0, 1, 3.3, or 10 mM cinnamate (Fig. 9C). The lactate-transport activity in MCT1b oocytes was completely inhibited by 1 mM cinnamate. In contrast, lactate-transport activity in MCT4b oocytes was not inhibited by 1 mM cinnamate but was partially inhibited by 3.3 or 10 mM cinnamate.

## Discussion

In the present study, we showed that spatially organized two lactate transporters facilitate O_2_ secretion from Root-effect hemoglobin by maintaining local blood pH low. We at first characterized the anatomical properties of the gas gland and rete mirabile in fugu swimbladder tissue at the light and electron microscopic levels. Those structures were well developed in the ventral wall, and we confirmed the presence of typical structures including basal labyrinth in gas gland cells and caveolae-like structures in endothelial cells of the rete mirabile (Figs. 1, 2). There are several advantages of using fugu for studying the mechanism of swimbladder O_2_ filling: its large size, well-developed gas gland and rete mirabile, and genome database availability. Similar studies are difficult to perform using small fish such as zebrafish or medaka. Pelster and Scheid [23] demonstrated that in eel gas gland tissue, the activity of enzymes involved in anaerobic glycolysis and the pentose phosphate shunt were as high as those in liver and white muscle, whereas the activity of enzymes responsible for oxidative metabolism in gas gland tissue was extremely low. Consistent with this notion, we observed high-level expression of GAPDH in fugu gas gland cells (Fig. 7B), indicating that (i) anti-GAPDH antibody can be used as a commercially available probe for identifying these cells and (ii) immuno- and *in situ* hybridization histochemistry of fugu swimbladder can demonstrate spatial organization of molecules involved in the O_2_-filling machinery.

As expected from previous studies that demonstrated the production of lactic acid in gas gland cells, we identified a monocarboxylate transporter, MCT4b, in the gas gland cells. MCT4b is thought to play a central role in the secretion of lactic acid from the gas gland cells to the blood vessels. On the other hand, it has been demonstrated, in cultured gas gland cells, that acid secretion was not inhibited by 1 mM cinnamate, an inhibitor of MCTs [15]. At first, this fact seemed to contradict our conclusion that lactate is secreted by MCT4b. Nevertheless, our kinetic analyses indicated that MCT4b is a low-affinity, high-capacity transporter and is not inhibited by 1 mM cinnamate (Fig. 9C), eliminating the apparent discrepancy. The low-affinity, high-capacity property of MCT4b to lactic acid may prevent from saturation of efflux where the local lactic-acid concentration is high. Cinnamate sensitivity of MCT4 in mammals is also low [24], suggesting that the characteristic is conserved from fish to mammals.

**Figure 10:**
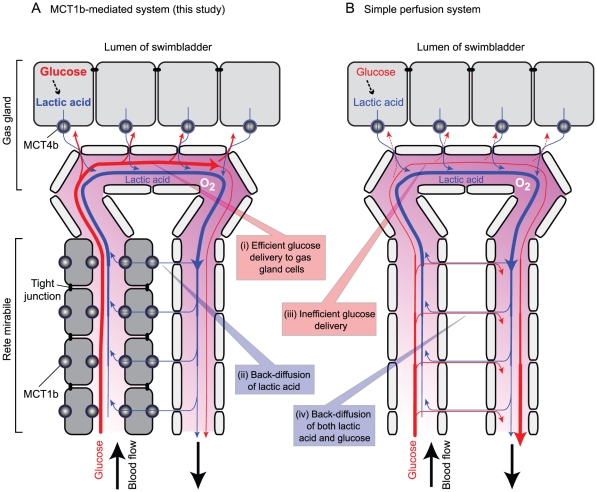
Roles of MCT1b and MCT4b in the fugu swimbladder. A, A model of lactic acid transfer mediated by MCT1b and MCT4b. In this model, the presence of MCT1b in tightly sealed arterial capillaries of the rete mirabile allows back-diffusion of lactate from the venous side to the arterial side (box ii), despite the presence of tight junctions that are essential for the efficient delivery of glucose to gas gland cells (box i), by preventing its shunt diffusion directly to the venous side (box iv). The expression of MCT1b and MCT4b in arterial capillaries and gas gland cells was demonstrated in this study. A loose (leaky) association between venous capillary endothelial cells and a tight (impermeable) adhesion between arterial capillary endothelial cells were demonstrated by Wagner [25], [26]. Gas gland cells' glucose requirement and lactic acid secretion have been demonstrated by others [14]. B, A model of the rete mirabile assuming free permeability between arterial and venous capillaries. In the absence of restricted permeability (tight junctions), delivery of glucose to gas gland cells becomes inefficient (box iii).

An unexpected finding was the presence of another high-affinity monocarboxylic acid transporter, MCT1b, in the arterial capillaries of the rete mirabile. Since the rete mirabile is a typical countercurrent system in which transport between inflow and the outflow is generally passive, the presence of the transporter MCT1b only on the inflow side was at first a puzzle. Considering that gas gland cells demand a large amount of glucose to generate lactic acid by glycolysis, we later realized that this localization of MCT1b is ideally suited for delivering glucose to the gas gland cells (Fig. 10). To prevent glucose loss from the inflow vessels (i.e., arterial capillaries) to the outflow vessels (venous capillaries) of the countercurrent system, the intercellular space among the endothelial cells of the arterial capillaries must be tightly sealed by tight junctions, which in turn become a barrier for recycling lactic acid through the paracellular pathway from the outflow to inflow vessels. However, this barrier could be circumvented by the transendothelial cell pathway mediated by MCT1b. This newly proposed model, diagrammed in Fig. 10, is supported by previous findings of tight junctions on arterial capillaries and a loose association between venous capillaries [25], as well as by a differential strength in light microscopy staining of actin filaments located on the cytoplasmic side of cell–cell adhesions (Fig. 8A). There is some physiological evidence suggesting the impermeability of the arterial capillaries in the rete mirabile. Wagner et al. [26] showed in a perfusion experiment that tannic acid did not permeate arterial capillary walls but did permeate the venous capillaries of the eel rete mirabile. Analyses of capillary permeability of isolated eel rete mirabile using radiolabeled water, urea, glucose, insulin, and albumin demonstrated that water is exchanged more rapidly than would be expected, in terms of its molecular size [19]. These results suggest that a significant barrier is present in arterial capillaries, and carriers mediate solute-specific transcellular permeation by the arterial capillaries. The rete mirabile has been a major focus of countercurrent system research and will become a useful model for further study of the O_2_ transport heterogeneity of the capillary endothelium [27], [28].

A similar system operates in the eyes of certain fish to support the metabolic needs of the retinal cells. High partial pressure of oxygen is maintained there by the Root effect and the countercurrent capillary network called the choroid rete mirabile, which has been known to exist in the fish for almost two centuries [29], [30] and to be evolved independently in several groups of teleosts [31]. The present study may also shed light on the mechanism how the choroid rete acts to maintain a relatively high pressure of oxygen.

## Materials and Methods

### Animals

Fugu (*Takifugu rubripes*) were purchased locally and kept in 150-liter tanks at 18–22°C. Artificial seawater (Rohtomarine) was obtained from Rei-Sea (Tokyo, Japan). All fugu were anesthetized by immersion in 0.1% ethyl *m*-aminobenzoate (MS-222, tricaine) before being sacrificed by decapitation. The animal protocols and procedures were approved by the Institutional Animal Care and Use Committee of Tokyo Institute of Technology (Fugu) and Mayo Clinic (*Xenopus*).

### Histological analyses

Fugu swimbladders were fixed in 4% paraformaldehyde in 0.1 M phosphate buffer, pH 7.4, embedded in paraffin, sectioned, and stained with hematoxylin and eosin according to standard procedures. Images were obtained with a microscope equipped with a digital charge-coupled device (CCD) camera (AxioCam HRc; Carl Zeiss, Oberkochen, Germany), and processed with AxioVision 4.1 software (Carl Zeiss).

### Transmission electron microscopy

Fugu were perfused with Hank's balanced salt solution (HBSS) under anesthesia through the ventral aorta. The swimbladders were removed, fixed with 4% (w/v) paraformaldehyde and 2.5% glutaraldehyde, and processed by standard procedures including tannic acid staining. Ultrathin sections were cut and examined with an electron microscope (Model H-7500; Hitachi, Tokyo, Japan).

### RNA isolation

Total RNA of fugu was isolated by acidic guanidinium thiocyanate–phenol–chloroform extraction with Isogen (Nippon Gene, Tokyo, Japan) as previously described [32]. The RNA was dissolved in diethyl pyrocarbonate–treated water, and its concentration was calculated from absorbance at 260 nm.

### Database search

To obtain the nucleotide sequences, BLAST searches were performed on the fugu genomic database (http://genome.jgi-psf.org/Takru4/Takru4.home.html) for *Takifugu rubripes*, and on Ensembl (http://www.ensembl.org/index.html) for *Danio rerio* and *Xenopus laevis*. To analyze the phylogenetic relationships of MCTs and SMCTs, the amino acid sequences were aligned using ClustalW software [33] and a phylogenetic tree was constructed using MEGA software [34] based on the neighbor-joining method with 2,000 bootstrap replicates. Synteny analysis was performed with Ensembl genome browser.

### Semiquantitative reverse transcription (RT)-PCR and molecular cloning

Five µg of total RNAs were reverse transcribed using oligo(dT) primers and the SuperScript III First-Strand Synthesis System (Invitrogen, Carlsbad, CA, USA). Primers were designed based on the genomic database (Table S1). After amplification, the reaction mixture was run on a 1.2% agarose gel, stained with ethidium bromide, and analyzed with a Kodak (Rochester, NY, USA) Image Station 2000R. Accession numbers of the sequences of the PCR products are as follows (“f” for “fugu”): fMCT2a, AB610610; fMCT2b, AB610611; fMCT2c, AB610612; fMCT5, AB610613; fMCT6, AB610614; fMCT7a, AB610615; fMCT7b, AB610616; fMCT8, AB610617; fMCT9, AB610623; fMCT12a, AB610618; fMCT12b, AB610619; fMCT13, AB610620; fSMCT1a, AB610621 and AB700117; fSMCT1b, AB610622, AB610625, and AB700118. Full-length cDNAs of fMCT1a, fMCT1b, fMCT4a, and fMCT4b were isolated from the swimbladder or other tissues by RT-PCR. cDNAs were directly sequenced or subcloned into pcDNA3 and then sequenced. The sequences have been deposited under GenBank/EMBL/DDBJ accession numbers AB574422, AB535599, AB610607, and AB574421.

### *In situ* hybridization

Swimbladders from fugu were perfused and fixed with 10% phosphate-buffered neutral formalin, harvested, embedded in paraffin, and sectioned (4 µm). A 262-bp fragment of fMCT1b cDNA (nucleotides 1100–1361), a 629-bp fragment of fMCT4b cDNA (nucleotides 727–1355), and a 445-bp fragment of fMCT1a cDNA (nucleotides 1020–1464) were used as DNA templates for the preparation of digoxigenin (DIG)-labeled riboprobes. DIG RNA labeling mix (Roche Diagnostics, Mannheim, Germany) was used to synthesize DIG- labeled sense and antisense probes. Alkaline phosphatase-conjugated anti-DIG antibodies and nitro blue tetrazolium/bromochloroindolyl phosphate substrates were used to visualize hybridization, followed by counterstaining with Kernechtrot (Muto Pure Chemicals). Images were obtained with a TOCO automatic virtual slide system (Claro, Hirosaki, Japan) and a microscope equipped with a digital CCD camera (AxioCam HRc; Carl Zeiss, Oberkochen, Germany), and processed with AxioVision 4.1 software (Carl Zeiss).

### Antibody production and specificity

Polyclonal antisera were made in rabbits that had been immunized with a keyhole limpet hemocyanin (KLH)-conjugated synthetic peptide corresponding to a part of MCT1b (amino acid residues 439–455) and MCT4b (amino acid residues 208–222 and 436–451). Antibody specificity was established by staining of COS7 cells exogenously expressing fugu MCT1b and MCT4b proteins as previously described [35]. COS7 cells were transfected with a pcDNA3.1 expression vector containing the fMCT1b and fMCT4b-coding sequences in its *Eco*RV site using Lipofectamine 2000 (Invitrogen). For immunofluorescence experiments, the cells were fixed with 4% paraformaldehyde (PFA) in PBS (pH 7.4) at 4°C for 10 min, permeabilized with 0.2% Triton X-100 in PBS at 4°C for 10 min, and blocked with 5% FBS in PBS for 1 h at room temperature. After blocking, cells were incubated with primary antibodies (1∶1,000 dilution) for 1 h at room temperature followed by Alexa Fluor 488-labeled secondary antibodies (Invitrogen) for 30 min at room temperature. Nuclei were stained with Hoechst 33342 (100 ng/ml). Fluorescence images were obtained with a laser confocal microscope (TCS-SPE; Leica, Wetzlar, Germany) using a fixed setting and processed with LAS AF software (Leica).

### Immunohistochemistry

Swimbladders from fugu were fixed in 4% PFA in 100 mM phosphate buffer (pH 7.4) at 4°C for 2 h, and rinsed in PBS. The tissues were cryoprotected through a series of increasing sucrose concentrations up to 20% and quick frozen in optimum cutting temperature compound (Sakura Finetek, Tokyo, Japan). Frozen sections (6 µm) were prepared, permeabilized with 0.2% Triton X-100 in PBS at room temperature for 10 min, and incubated with 5% FBS (Invitrogen) in PBS at room temperature for 1 h. After blocking, the sections were incubated with anti-fMCT1b antiserum (1∶1,000), anti-fMCT4b antiserum (1∶5,000), and anti-GAPDH antiserum (1∶100, Sigma) in PBS containing 5% FBS at 20°C for 16 h. After washing with PBS, the sections were incubated with a mixture of Alexa Fluor 488-labeled secondary antibodies (1∶2,000), Alexa Fluor 594-labeled ConA (50 µg/ml; Invitrogen), and Hoechst 33342 (100 ng/ml) in PBS containing 5% FBS at 20°C for 1 h. The sections were mounted on antifade glycerol (90% glycerol, 10% 10× PBS, and 0.1% 1,4-phenylenediamine at pH 7.4). Fluorescence images were obtained as described above.

### Expression and electrophysiology of fMCT1b and fMCT4b in *Xenopus* oocytes

The entire coding regions of fMCT1b and fMCT4b cDNAs were inserted to the pGEMHE *Xenopus laevis* expression vector [36], [37]. The plasmids were linearized with *Not*I, and cRNAs were transcribed in vitro using the T7 mMESSAGE mMACHINE kit (Ambion, Austin, TX, USA). *X. laevis* oocytes were dissociated with collagenase as previously described [38] and injected with 25 nl of water or a solution containing cRNA at 0.4 g/L (10 ng/oocyte). Oocytes were incubated at 16°C in filtrated ND96 media, and studied 7–10 days after injection. ND96 contained 96 mM NaCl, 2 mM KCl, 1 mM MgCl2, 1.8 mM CaCl2, and 5 mM HEPES (pH 7.5).

To measure intracellular pH (pH_i_), a H^+^ ion-selective microelectrode was prepared with an H^+^ ionophore I-mixture B ion-selective resin (Fluka Chemical, Ronkonkoma, NY), and used as previously described [38]. pH_i_ was measured as the difference between a pH electrode and a KCl voltage electrode impaled into the oocyte, and membrane potential (*V*_m_) was measured as the difference between a KCl microelectrode and an extracellular calomel [38]. pH electrodes were calibrated using pH 6.0 and 8.0 buffers (Fisher), followed by a point calibration in ND96 (pH 7.50) as described previously [38].

H^+^/lactate cotransport activities were measured as changes of pH_i_ as follows. An oocyte was perfused with ND96 solution and then perfused with ND96 containing 70 mM Cl^−^ (70Cl-ND96). Thirty-three mM NaCl of ND96 was replaced with 33 mM sodium gluconate, and named 70Cl-ND96 (ND96 containing 70.6 mM Cl^−^). After that, the oocyte was perfused with 70Cl-ND96 containing lactic acid. To prepare 70Cl-ND96 containing 33 mM lactic acid, 33 mM NaCl was replaced with 33 mM lactic acid and then the solution was titrated to pH 7.5 using NaOH. Test solutions with differing lactic acid concentrations were prepared by mixing 70Cl-ND96 with 70Cl-ND96 containing 33 mM lactic acid followed by titration to pH 7.5. Lactate-elicited changes in pH_i_ were measured by addition of 1, 3.3, 10, or 33 mM lactic acid in 70Cl-ND96, and initial rates of pH_i_ per second were calculated. Lactate steady-state kinetics were calculated based on the Michaelis–Menten equation by using GraphPad Prism software (Prism 5.0, GraphPad, San Diego, CA, USA).

Inhibition of MCT1b and MCT4b by cinnamate was tested as follows. To prepare 70Cl-ND96 containing 33 mM cinnamic acid, 33 mM NaCl was replaced with 33 mM α-cyano-4-hydroxycinnamic acid (Sigma) and then the solution was titrated to pH 7.5 using NaOH. Test solutions with differing concentrations of cinnamate and lactate were prepared by mixing 70Cl-ND96 with 70Cl-ND96 containing 33 mM cinnamic acid and 70Cl-ND96 containing 33 mM lactic acid followed by titration to pH 7.5. An oocyte was perfused with 70Cl-ND96 containing cinnamic acid and incubated for 10 min. After that, the oocyte was perfused with 70Cl-ND96 containing cinnamic acid and 10 mM lactic acid. Lactate-elicited changes of pH_i_ in the presence of 1, 3.3, or 10 mM cinamic acid were measured, and initial rates of ΔpH_i_/s were calculated.

## Supporting Information

Figure S1 **Functional and anatomical bases for generating and forcing oxygen into the swimbladder.**
(EPS)

Figure S2 **Multiple alignment of amino acid sequences of MCT1 (A) and MCT4 (B) families.**
(EPS)

Figure S3 **Specificity of anti-MCT1b and anti-MCT4b antibodies.**
(TIF)

Table S1 **List of primers used for PCR amplification.**
(DOCX)
