# Supplementary figures and images for: O2-Filled Swimbladder Employs Monocarboxylate Transporters for the Generation of O2 by Lactate-Induced Root Effect Hemoglobin
Source: PLoS One. 2012 Apr 4;7(4):e34579. doi: 10.1371/journal.pone.0034579 (PMC3319611; doi:10.1371/journal.pone.0034579)

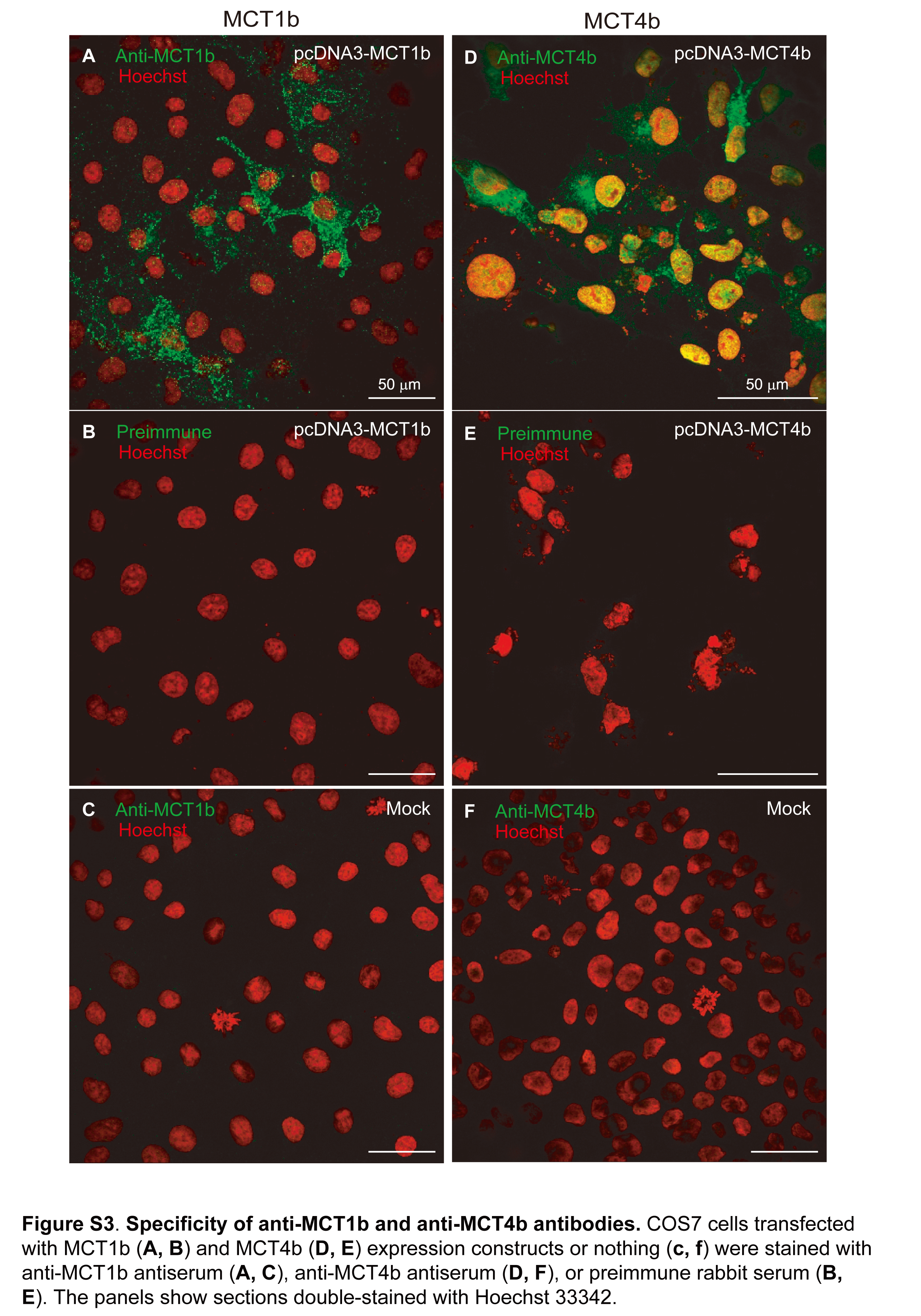

Supplement: Figure S3 — Specificity of anti-MCT1b and anti-MCT4b antibodies. (TIF) [file pone.0034579.s003.tif]
