# Supplementary material for: O2-Filled Swimbladder Employs Monocarboxylate Transporters for the Generation of O2 by Lactate-Induced Root Effect Hemoglobin
Source: PLoS One. 2012 Apr 4;7(4):e34579. doi: 10.1371/journal.pone.0034579 (PMC3319611; doi:10.1371/journal.pone.0034579)
Supplement: Table S1 — List of primers used for PCR amplification. (DOCX) [file pone.0034579.s004.docx]

| **Table S1. List of primers used for PCR amplification.** | | | |
| --- | --- | --- | --- |
| Gene | Sequence | Remarks | |
| fMCT1a | CCGCTCTCCTCTTGTCCATC | RT-PCR (S) | |
|  | GATGATGCCAGAGGAGATGTAG | RT-PCR (AS) | |
|  | GTATTTTTCCTCTCTGCTGTTTCTC | cDNA cloning (S) | |
|  | GGCTTCTCTATAGATAACTCGGTG | cDNA cloning (AS) | |
| fMCT1b | CAAAAACCACAGAGAGGAGGAC | RT-PCR (S) | |
|  | AGTCGGTTTGGCAACATTGTC | RT-PCR (AS) | |
|  | GACGAGGTTTTGTGAGCGAC | cDNA cloning (S) | |
|  | TGGCATTGCATGGTACAGAG | cDNA cloning (AS) | |
| fMCT2a | CACCACGAAAGAGAAGCTGAC | RT-PCR (S) | |
|  | TGTCTCAGTCCGTTTGTACATC | RT-PCR (AS) | |
| fMCT2b | CTTCCTTCGGCACCTCTATC | RT-PCR (S) | |
|  | AGGCGCAAAGAACCCAAAGAAC | RT-PCR (AS) | |
| fMCT2c | ACTGGTCAATCGCTACGGATG | RT-PCR (S) | |
|  | CAGGAATTGAGCATCAGCCCT | RT-PCR (AS) | |
| fMCT4a | CCAAGCTTCTGGACTTCTCTG | RT-PCR (S) | |
|  | CTGGAGTTGGGGACTTCTTG | RT-PCR (AS) | |
|  | AAGCAGGATGGGAGGTGTAGCGTTG | cDNA cloning (S) | |
|  | CTGATCACGTGTCACTTCAACATACTGG | cDNA cloning (AS) | |
| fMCT4b | GGCCTCTTTTTCCACGAGCAT | RT-PCR (S) | |
|  | GTTGACCACAAATACAGGAGGTAC | RT-PCR (AS) | |
|  | CAGAAAGCATCATGGGAGGAG | cDNA cloning (S) | |
|  | TGTGGGAAAAGAGTTGTGTAACT | cDNA cloning (AS) | |
| fMCT5 | TGCATCTACACCTGGTCACTG | RT-PCR (S) | |
|  | ATGGAGTCGTTGACCCTTTC | RT-PCR (AS) | |
| fMCT6 | AAGGACGCCATGTTTATGTG | RT-PCR (S) | |
|  | AAAGACACGATGAGGAAAATG | RT-PCR (AS) | |
| fMCT7a | CAAAGTGAGCTGCAGGAAAAC | RT-PCR (S) | |
|  | GCTCCATAATTGTGAGTGACATC | RT-PCR (AS) | |
| fMCT7c | ATTCTCTCTCTGTACTTCACCCGT | RT-PCR (S) | |
|  | AGCTTTGTGTTTCGGCACCTTTG | RT-PCR (AS) | |
| fMCT8 | GGACACTAAAAAGGAATGGGTGC | RT-PCR (S) | |
|  | AACACGCACACGACCACCA | RT-PCR (AS) | |
| fMCT9 | TCACATGTCAGTACTTTGAGAAGAG | RT-PCR (S) | |
|  | CACATCTTCAATAAAGAGCACAG | RT-PCR (AS) | |
| fMCT12a | TTTCCTTGGGAACTGGCTCTC | RT-PCR (S) | |
|  | ACACAGGTTGGAAACCAATCCTC | RT-PCR (AS) | |
| fMCT12b | CTCCATCTTCTTTGTGGAGTTTCAG | RT-PCR (S) | |
|  | CACCACACACACACAGGTTG | RT-PCR (AS) | |
| fMCT13 | TGGGCATCACTTTCATCAAC | RT-PCR (S) | |
|  | TGAAGGAAGCCACGTAGTTG | RT-PCR (AS) | |
| fTAT1 | ACGTGGAGGAGCGTTTTGGACCAGA | RT-PCR (S) | |
|  | TGCCAGGTAGAAGGCCACGTCGTAG | RT-PCR (AS) | |
| fSMCT1 (fSMCT1a) | AACCCGTCTGCTGGGGACT | RT-PCR (S) | |
|  | AGACCGAGTACAGACACATTCC | RT-PCR (AS) | |
|  | CACCAAAATGTCAGGGGACGCTGGGGTTGG | cDNA cloning (S) | |
|  | AACAAAATTAGTGTGAGTTCATGTTGCTCT | cDNA cloning (AS) | |
| fSMCTe (fSMCT1b) | ACGGTGTCTTCAAGCATTAATG | RT-PCR (S) | |
|  | GACAGCAAGGTAAAGATAGGACAG | RT-PCR (AS) | |
|  | AGGAAACATGGTTGGGACAGAGGCCACATT | cDNA cloning (S) | |
|  | GGTAGCTTTAGAAATGGGTGACTGCTTCAG | cDNA cloning (AS) | |
| actin | AGCGTGGGTACTCCTTCACTAC | RT-PCR (S) | |
|  | TCGTACTCCTGCTTGCTGATCC | RT-PCR (AS) |  |

S, sense primer; AS, antisense primer.
